# Some Uses of Nitro-Glycerine

**Published:** 1884-03

**Authors:** Charles G. Stockton

**Affiliations:** Professor of Materia Medica, Niagara Medical College


BUFFALO

Medical and Surgical Journal.

Vol. XXIII. MARCH, 1884. No. 8.

### Original Communications.

### Some Uses of Nitro-Glycerine.*

By Charles G. Stockton, M. D.,
Professor of Materia Medica, Niagara Medical College.

*Mr. President and Gentlemen of the Medical Club:*

Those among you who had the privilege of listening to the instructive paper on angina pectoris, read before this body at its meeting, Nov. 9, 1881, by our associate, Dr. Hopkins, heard the following :†

"The pulse, as you would suspect, is always changed, but in a way that requires critical examination for its detection. My observation is that the principal alteration in the pulse is in its volume. In patients with whose circulation we are reasonably familiar, we see that the pulse, instead of being full and soft, is small and resisting, and tells at once of a contracted artery." Again, "upon the theory that vaso-motor spasm exists as a factor of angina pectoris, a remedy which acts by relieving the spasm suggests itself as being directly curative. All the cases I have seen published go to show that when given in angina, nitro-glycerine acts uniformly and beneficially. I have given

* Read before the Medical Club, February, 1884.

† Buffalo Medical and Surgical Journal, Dec., 1881.

it in several cases of lead colic, and have found the high arterial tension peculiar to that condition to be relieved, and that with this improvement, the pain and constipation also disappear."

After reading the articles in the London *Lancet* for 1879, to which Dr. Hopkins referred, I felt convinced that we possessed in nitro-glycerine an agent with which we might successfully oppose arterial tension that results so frequently from varied pathological conditions.

Accordingly, the drug was used as opportunity afforded, and part of my experience I purpose reading to you to-night.

Observers are in accord that the action of nitro-glycerine resembles closely that of its congener, amyl nitrite. They both produce remarkable arterial relaxation—a chocolate-like color of arterial blood, a depression of the motor nerve centres and the pneumo-gastric, and muscular paresis.

Nitro-glycerine departs from the similarity in this, that it is slower and more prolonged in its action; it does not act as positively to lower animal temperature; the resulting arterial relaxation and flushing of the face is not so pronounced as is the case with nitrite of amyl. Without speculating over all the physiological actions of nitro-glycerine, it would perhaps be profitable to formulate our knowledge, as regards its effects upon the circulatory system.

1st. It has a direct paralyzing effect upon the muscular coat of the blood vessels, especially the capillaries, producing relaxation of these, widening of the area vasculosa, thereby directly lessening arterial tension.

2d. While this action is peripheral, it undoubtedly is assisted by a similar accompanying centric influence, from depression of the vaso-motor centres.

3d. There is depression of the inhibitory powers of the pneumo-gastrics, and, as a result, the heart beats more rapidly while respiration becomes slower, death ensuing from asphyxia.

To state it in another way, we have from the influence of this agent great vascular relaxation, with increase of heart action, the efforts of the heart arising, partly, from the sudden capillary dilatation, the increased blood supply to those vessels making the heart labor to supply itself, and partly from the loss of the inhibitory function of the pneumo-gastrics.

Nitro-glycerine appears to be quite uniform and constant in its manifestations. You are aware how successful it proves in angina pectoris, and it has been employed with gratifying results in spasmodic asthma, bronchitis, sea-sickness, reflex-vomiting, whooping cough, migraine, epilepsy, the cold stage of intermittents, acute and chronic Bright's disease, and numerous other affections.

About two years ago I first gave the drug to a patient whose case I shall briefly describe.

*Case* 1. Miss H. is a laundress, aged 30 years, a hard worker, and one having much responsibility. Nine years since she began to have pain over the apex of the heart. Sometimes this continued for a few days, sometimes for weeks. She became progressively worse. After three years the left arm, shoulder and finally the side became numb,—sensation was impaired in the parts.

The pain was associated with dyspnœa. The seizures were occasionally abrupt. She fell to the ground senseless and pulseless. Stimulants always afforded temporary and limited relief.

She received nitro-glycerine, ♏i of a one per cent. solution in alcohol. The dose was increased, day by day, until she took ♏iij t. i. d., and was promptly tranquilized and relieved from pain. Afterwards she took ♏i at night for a certain period of time, and called herself well. Eventually the disturbance returned and she took at last ♏vii at one dose with good effect.

Her bottle becoming empty, she was given an order to repeat the prescription, did so, and of the new preparation took ♏vii—and then concluded that her end was at hand.

The face was flushed, turgid, eyes suffused, cardiac pulsations forcibly felt throughout the body, she was weak, almost without muscular control, but the most prominent disorder was cerebral.

She said she "thought the top of her head was coming off, and could feel it jerk up with every throb of the heart."

In this case there is no sensible lesion. I have heard at times a slight pre-systolic murmur, which was not evident when the attack subsided. The spine is irritable, likewise the track of the fifth and sixth intercostal nerves, otherwise the woman is healthy. How may we account for the different strengths of the solutions taken? I satisfied myself that the patient was not deceived in the dose. The pharmacist said that the last bottle was filled from a new package from another druggist, marked the one per cent. solution. Now I have discovered that there are in the shops two preparations, one of which is the one per cent. and the the other the ten per cent. solution, and I shall always believe that my patient had the latter, and took seven-tenths of a grain of nitro-glycerine at a dose, the equivalent to seventy ♏ of the common solution. This appears to me as a very important matter.

*Case* 2. About this time, at the Church Home, was an old lady of over 90 years, who nourished poorly, was constipated, suffered from pains all over the body, had dyspnœa, was unable to sleep quietly, and was very unhappy. There was marked arterial tension, and slow pulse. An extensive arcus senilis was present. The condition of the heart I have forgotten, for of this case I have no notes. She was given ♏j of the one per ct. sol. of nitro-glycerine, and was improved. The pulse became softer and quicker, she ate, slept, and expressed herself as feeling better than for a long time. During the remainder of her days she had occasional recourse to nitro-glycerine, and always with benefit.

*Case* 3. Soon after, I was invited to prescribe for Mr. P., an old gentleman of 83 winters, an American by birth. I learned that he had felt as well as usual upon going to bed the night before, but when wakened in the morning, upon attempting to rise, he fell back fainting, and remained in a semi-unconscious condition until my arrival. I found him with a somewhat weak but tense pulse, the radial arteries were felt to be hard under pressure, showing unmistakable signs of degeneration. The heart sounds were feeble. There was a systolic murmur, probably aortic in origin; the mitral valves seemed to me intact. He exhibited a most remarkable arcus senilis, and undoubtedly suffered from a form of vascular degeneration of old people; the immediate cause of my summons being an attack of syncope, depending upon his weakened heart and narrowing of his cerebral blood vessels. Having given some stimulants, which his stomach was not disposed to retain, I sent for and administered ♏ij of one pr. ct. sol. of nitro-glycerine, with the happiest result. He felt better immediately. The following day he was able to sit up a little, but continued his remedy in ♏j doses, for a few days. A week after, he walked six blocks to my office without great fatigue. His pulse was full and softer, his expression was good, and he was as loquacious as usual. He stated that he had discontinued his medicine one day, but feeling his old symptoms returning, again resorted to it with the former result.

Nine months afterwards my patient was in usual health, and had taken none of his remedy for a long time. The question may be asked, would not stimulants and tonics have acted as well? Possibly, but they could have acted no better.

The last two cases referred to were lean old people; my next case was a fat old woman; and it seems to me worth while to pause here and emphasize, by considering the probable conditions of these patients.

Sir James Paget speaks of the "withering and the fatty degenerations."

He says: "Some people, as they grow old, seem only to wither and dry up; sharp featured, shriveled, spinous, old folk, yet withal, wiry and tough, clinging to life, and letting death have them, as it were, by installments, slowly paid. Such are the lean and slippered pantaloons; and their shrunk shanks declare the pervading atrophy. Others, women more often than men, as old, and as ill-nourished as these, yet make a far different appearance. With these, the first sign of old age is that they grow fat; and this abides with them till, it may be, in a last illness, sharper than old age, they are robbed even of their fat. These, too, when old age sets in, become pursy, short-winded, pot-bellied, pale and flabby; their skin hangs not in wrinkles but in rolls; and their voices, instead of rising 'towards a childish treble,' become gruff and husky."*

I have quoted these lines because the great surgeon has described, better than I could hope to, those types of cases in which I have had the most experience and success in the use of nitro-glycerine.

It is unnecessary to remind the members of this body, that in the onward march of degenerations, vascular atrophies often occupy the front rank; that these progress until the vessels are unequal to meet even the "diminished requirements of nutrition." Is it not largely owing to this lessened nutrition, this tissue starvation, that we have the pains, the miseries, the unhappiness so constant with the aged? And if this is so, may we not, by the use of an agent that relaxes the tension of these degenerated vessels, allowing the blood to flow easily and abundantly through its courses to the sterile tissues, alleviate many of the sufferings and make smoother the downward path to the grave of those who exclaim with Lear:

> "You see me here, you gods, a poor old man,
> As full of grief as age; wretched in both?"

These thoughts are suggested, because I have had occasion to notice that such cases as those above described are never done telling of their ailments; and I have observed that nitro-glycerine, in some instances, has added much to the comfort, if it has not prolonged the life of these people, by helping the organs to maintain a condition more consistent with the exercise of their functions.

* Paget's Surgical Pathology, p. 82.

*Case* 4. Celia P., an English woman, aged 80 years; a typical case of fatty degeneration. I think her weight was considerably over two hundred pounds, and she was not tall. Having labored down stairs to her dinner one day, she fainted. I found her half an hour later, pulseless, cold, gasping, unable to speak. Stimulants were administered; later, digitalis and strychnine. She rallied a little, and with the assistance of two strong men, I got her to bed. It was with the greatest difficulty that life was maintained by means of the remedies above mentioned. After the lapse of several days, she was given nitro-glycerine. The improvement was prompt, decided and progressive. She enjoyed her life for some months afterwards, but only with the use of her medicine. Whenever it was discontinued, the patient quickly grew worse, nor could we supply the place of nitro-glycerine. I should like to recite the history of several other cases almost precisely like the foregoing, but I hesitate to occupy your time. I am positive that none of our usual remedies possess such conspicuous advantages as this in the treatment of such cases as I have described. In the hospital of the Erie County Penitentiary, I tried the effects of nitro-glycerine in a variety of cases with varying results.

*Case* 5. Pat. D., alias "Paddy the Pig," about 50 years of age, man of all work in a second-class hotel. Had had a hard spree, and came in poorly nourished and somewhat frenzied. His face and lower extremities were very œdematous, expression cyanotic, much dyspnœa, evidently general venous engorgement present. Area of precordial dullness excessive. There was a loud systolic murmur over the aortic valves, which seemed to depend upon regurgitation. He was treated in the usual way, and made general improvement, except as regarded the circulatory system. After the lapse of a week he was given nitro-glycerine, one drop of a ten per cent. solution. Almost immediately he became partly deaf, but his dyspnœa was relieved, and the arterial and venous circulations seemed pretty well balanced. After much difficulty, a specimen of urine was obtained. It was found to contain traces of albumen, the specific gravity was 1.025. Paddy's anasarca did not improve, although his appetite did. In addition to the foregoing treatment, he was given the fluid extract of convallaria, without benefit. At last, tonics, with bandaging of legs, relieved his dropsy, and when discharged, three weeks later, his heart sounds were almost normal. His deafness disappeared with the discontinuance of the nitro-glycerine.

*Case* 6. To another man suffering from dilatation of the heart, with supposed aortic stenosis and mitral insufficiency, having a small, irregular, compressible pulse, I gave nitro-glycerine, with bad results. After the third dose of three minims of the one per cent. solution, he suffered so from dyspnœa, headache and general distress, and the heart was so tumultuous in its action, that the drug was withdrawn.

*Case* 7. To a man of sixty, who appeared to have pyelitis (which diagnosis was afterwards disproved by the microscope and who really suffered, I suppose, from urethral fever), I gave nitro-glycerine to relieve arterial tension. This it accomplished, indeed, but with so much prostration, accompanied with vomiting and hiccough, that the treatment was changed.

*Case* 8. Myalgia, accompanied with weak heart, hot head, cold extremities and impaired digestion, was benefited by nitro-glycerine.

*Case* 9. One of seminal emissions with a weak, intermittent and excitable pulse, was not improved by the drug. His face flushed, his head ached, he felt miserable. There were numerous other similar experiments made, in which it was hoped that, by relieving the opposition to the arterial current, by relaxing the capillaries, the heart action would become less labored, nutrition improved and functions restored. I have seen no real good from nitro-glycerine in migraine, although I have employed it in three cases that I thought well selected. Hammond, before the New York Neurological Society, Oct. 4, 1881, eulogized the drug, in what he termed, the "angio-spastic form of migraine," and stated that it would be contra-indicated in "the angio-paralytic form." Perhaps my cases were "angio-paralytic." But the doctor refers to a case which was "angio-spastic" on the one side, and "angio-paralytic" on the other, cured almost instantly, after a long failure with other treatment. Paradoxes are excusable in nervous diseases. Dr. Hammond also states that in status epilepticus, and some other forms of epilepsy, when bromides are not efficient, nitro-glycerine is very effective.*

The use of the drug in epilepsy was first suggested, I believe, by Mr. Robson, F. R. C. S., Leeds, in the *British Medical Journal*, April 10, 1880. This gentleman also reported a series of eight interesting cases of chronic Bright's disease, and of others, accompanied by that condition of the vessels which was described by Sir Wm. Gull and Dr. Sutton. In speaking of the arterial tension present in these cases, he says: "Whether it be due to chronic kidney mischief, or to arterial fibrosis, this condition is unquestionably relieved by nitro-glycerine, and, with the diminution of pressure, in my experience, improvement invariably follows, though, in some cases, it may only be temporary.† ‡ Mr. Robson also suggests the use of nitro-glycerine in the vascular tension of the aged. Dr. Turnbull and others have reported favorably of its effects in sea-sickness; various authorities praise its action in asthma and neuralgia. This drug, which has been before the profession for over twenty years, has received, I venture to assert, less than its deserved recognition. Perhaps this has in part arisen from its well-known dangerous properties in an undiluted form. It is said to be perfectly safe in an alcoholic or ethereal solution, or in chocolate tablets, or pills. The latter have to be chewed before swallowing, it is stated, else their activity is liable to remain latent. It was suggested by some wag that, before leaving packages of this medicine with a patient, they

* New York Medical Record, Oct. 22, 1881. Virginia Medical Monthly, Oct., 1881.
† British Medical Journal, Nov. 20, 1880
‡ Boston Medical and Surgical Journal, Jan. 10, 1884.

should be labeled, "*explosive, with care, containing dynamite.*" To summarize, I think I can safely say that the proper administration of nitro-glycerine uniformly produces vascular relaxation; and as vascular tension is a prominent factor in diseases of the circulatory and nervous systems, the drug is destined to a much more extensive use than it now enjoys. From my own experience I know it to be especially applicable in abbreviating or preventing attacks of angina pectoris, in restoring the normal vascular resistance in kidney diseases, and in alleviating many distressing symptoms of arterial degeneration in the aged.

